# Comparative expression patterns and diagnostic efficacies of SR splicing factors and HNRNPA1 in gastric and colorectal cancer

**DOI:** 10.1186/s12885-016-2387-x

**Published:** 2016-06-10

**Authors:** Won Cheol Park, Hak-Ryul Kim, Dong Baek Kang, Jae-Suk Ryu, Keum-Ha Choi, Gyeong-Ok Lee, Ki Jung Yun, Keun Young Kim, Raekil Park, Kwon-Ha Yoon, Ji-Hyun Cho, Young-Jin Lee, Soo-Cheon Chae, Min-Cheol Park, Do-Sim Park

**Affiliations:** Department of Surgery, School of Medicine, Wonkwang University, Iksan, South Korea; Department of Internal Medicine, School of Medicine, Wonkwang University, Iksan, South Korea; Department of Laboratory Medicine, School of Medicine, Wonkwang University, 895 Muwang-ro, Iksan, 54538 Republic of Korea; Center for Metabolic Function Regulation, Institute of Wonkwang Medical Science and Institute of Wonkwang Clinical Medicine, School of Medicine, Wonkwang University, Iksan, Korea; Department of Pathology, School of Medicine, Wonkwang University, Iksan, Korea; Department of Biomedical Science and Engineering, Gwangju Institute of Science and Technology (GIST), Gwangu, Korea; Department of Radiology, School of Medicine, Wonkwang University, Iksan, Korea; Department of Herbology, School of Oriental Medicine, Wonkwang University, Iksan, Korea

**Keywords:** Gastric cancer, Splicing factor, HNRNPA1, SRSF7, Carcinoembryonic antigen

## Abstract

**Background:**

Serine/arginine-rich splicing factors (SRSFs) and HNRNPA1 have oncogenic properties. However, their proteomic expressions and practical priority in gastric cancer (GC) and colorectal cancer (CRC) are mostly unknown. To apply SFs in clinics, effective marker selection and characterization of properties in the target organ are essential.

**Methods:**

We concurrently analyzed SRSF1, 3, and 5–7, and HNRNPA1, together with the conventional tumor marker carcinoembryonic antigen (CEA), in stomach and colorectal tissue samples (*n* = 420) using semiquantitative immunoblot, subcellular fractionation, and quantitative real-time polymerase chain reaction methods.

**Results:**

In the semiquantitative immunoblot analysis, HNRNPA1 and SRSF7 levels were significantly higher in GC than in gastric normal mucosa, and SRSF7 levels were higher in intestinal-type compared with diffuse-type of gastric adenocarcinoma. Of the SFs, only HNRNPA1 presented greater than 50 % upregulation (cancer/normal mucosa > 2-fold) incidences and CEA-comparable, acceptable (>70 %) detection accuracy (74 %) for GC. All SF protein levels were significantly higher in CRC than in colorectal normal mucosa, and HNRNPA1 levels were higher in low-stage CRC compared with high-stage CRC. Among the SFs, HNRNPA1 and SRSF3 presented the two highest upregulation incidences (88 % and 74 %, respectively) and detection accuracy (90 % and 84 %, respectively) for CRC. The detection accuracy of HNRNPA1 was comparable to that of CEA in low (≤ II)-stage CRC but was inferior to that of CEA in high (>II)-stage CRC. Extranuclear distributions of HNRNPA1 and SRSF6 (cytosol/microsome) differed from those of other SRSFs (membrane/organelle) in both cancers. In an analysis of the six SF mRNAs, all mRNAs presented unacceptable detection accuracies (≤70 %) in both cancers, and all mRNAs except *SRSF6* were disproportionate to the corresponding protein levels in GC.

**Conclusion:**

Our results provide a comprehensive insight into the six SF expression profiles in GC and indicate that, among the SFs, HNRNPA1, but not *HNRNPA1* mRNA, is the most effective, novel GC marker. Regardless of the good to excellent detection accuracy of SRSF3 and HNRNPA1 in CRC, the SFs have lower practical priority than CEA, especially for high-stage CRC detection.

## Background

Alternative splicing is a ubiquitous post-transcriptional process that leads to proteomic diversity and the disruption of splicing regulatory networks is a critical component of carcinogenesis.

The serine/arginine (SR) protein family is an important class of splicing regulators and its members, including SR splicing factor (SF) 1, SRSF3, and SRSF6, have shown multiple proto-oncogenic properties and aberrant expressions in various cancer cells [1–4]. SRSF1, 3, and 7 shuttle between nucleus and cytoplasm, and their subcellular distribution is associated with various cellular functions and reactive responses [5–7]. For instance, in the nuclear compartment of cells, SFs promotes the splicing process, while in the extranuclear compartment, they regulate protein translation [5]. In this sense, SFs are found in ribosomes, the translation machinery [5], and are expected to colocalize with SF-binding translation regulatory proteins such as the target of rapamycin complex 1 (TORC1), which locates in lysosomes or in cytosol [6]. Additionally, specific cellular stresses or conditions induce post-translational modifications of SFs which associated with cytoplasmic localization and the stability of SFs and/or inhibit general splicing process in nucleus [5–8].

Heterogeneous nuclear ribonucleoprotein A1 (HNRNPA1) is also a well-known splicing regulator with effects antagonistic to SR proteins [9]. Upregulated expression and aberrant cytoplasmic localization of HNRNPA1, as determined by immunohistochemical staining, were noted in colorectal cancer (CRC) [10]. Recently, HNRNPA1 has emerged as a plausible biomarker of CRC [11].

However, in gastric cancer (GC), proteomic expressions of these SFs are unclear and their diagnostic values have not been determined. The comparative practical priority among the SR proteins and conventional tumor markers in GC and CRC is unknown. Moreover, it is unclear as to whether the expression patterns and practical priority of these SFs differ between the cancers.

We aimed to: (1) identify the translational and transcriptional profiles of five SRSFs (SRSF1, SRSF3, SRSF5, SRSF6, and SRSF7) and HNRNPA1 in GC; and (2) compare the detection accuracy (DA) among the SFs and the currently used tumor marker carcinoembryonic antigen (CEA) [12] and determine specific features in these parameters in GC and CRC.

## Methods

### Subjects and sample preparation

A total of 420 fresh stomach and colon biopsy samples were obtained from 224 patients (Table 1) who had undergone surgical resection.

**Table 1:** Demographic characteristics of subjects and tumors

	Stomach (*n* = 147)			Colon and rectum (*n* = 273)		
			SF^a^ (*p* ^a^)			SF^a^ (*p* ^a^)
Total (*n* = 420)			Median^a^ (IQR)			Median^a^ (IQR)
Sample constitution						
Cancer	60			123		
Adenoma	–			15		
NM	87			135		
Sample pairing, *n*	(GC/NM)			(CRC/adenoma/NM)		
Doublet^b^	60 (60/60)			120 (113/7/120)		
Triplet^c^	–			8 (8/8/8)		
Unpaired (singlet)	27 (0/27)			9 (2/0/7)		
Subjects (*n* = 224), *n*	87			137		
Age, mean (range), years	66 (40–86)		NS-all SFs	69 (45–90)		NS-all SFs
≥65 years, *n* (%)	32 (53)			87 (71)		
Gender, male, *n* (%)	90 (61)		NS-all SFs	193 (71)		NS-all SFs
Type of cancer, *n*						
	AC	56		AC	120	
	Other^d^	4		Other^e^	3	
Location of AC, *n* (%)			NS-all SFs			NS-all SFs
	Cardia	5 (9)		Proximal^f^	29 (24)	
	Body	13 (23)		Distal^f^	63 (53)	
	Antrum	38 (68)		Rectum	28 (23)	
Histologic feature of AC, *n* (%)	Type		SRSF7 (0.049)	Differentiation		HNRNPA1 (0.020)
	Intestinal	44 (79)	7.0 (2.0–29.0)	Well	37 (31)	7.0 (4.2–11.0)
	Diffuse^g^	12 (21)	2.9 (1.9–4.8)	Moderate	83 (69)	5.0 (2.1–8.0)
				and poor		
Tumor status of AC, *n* (%)			NS-all SFs			NS-all SFs
T1, T2	27 (48)		–	19 (16)		–
T3, T4	29 (52)		–	101 (84)		–
LN metastasis of AC, *n* (%)			NS-all SFs			HNRNPA1 (0.003)
N0	28 (50)		–	62 (52)		7.0 (4.0–10.0)
N1, N2, N3	28 (50)		–	58 (48)		4.3 (1.9–7.5)
TNM stage^h^ of AC, *n* (%)			NS-all SFs			HNRNPA1 (0.003)
I, II	40 (71)		–	62 (52)		7.0 (4.0–10.0)
III, IV	16 (29)		–	58 (48)		4.3 (1.9–7.5)
Aneuploidy status of AC, *n* (%)			NS-all SFs			NS-all SFs
≤ 3 %	50 (91)		–	72 (66)		–
> 3 %	5 (9)		–	37 (34)		–

*Abbreviations*: *AC* adenocarcinoma, *CRC* colorectal cancer, *CEA* carcinoembryonic antigen, *GC* gastric cancer, *IQR* interquartile range, *LN* lymph node, *NM* normal mucosa, *NS-all SFs* not significant (*p* >0.05) for all splicing factor proteins and their mRNAs; SF = splicing factor

^a^SF name, *p* values, and median relative band intensity were described only for SF proteins or mRNAs that had a *p* value less than 0.05 in the six SFs; *p* values were acquired by Mann-Whitney *U*-test (between two groups) or Kruskal-Wallis test

^b^Two samples (cancer with its adjacent NM or adenoma with its adjacent NM) acquired from the same patient

^c^Three samples (cancer, adenoma, and their adjacent NM) were acquired from the same patient

^d^Gastrointestinal stromal tumor (*n* = 3) and neuroendocrine carcinoma (*n* = 1)

^e^Lymphoma (*n* = 2) and malignant melanoma (*n* = 1)

^f^Proximal colon indicates cecum, ascending colon, and transverse colon. Distal colon indicates splenic flexure, descending colon, sigmoid colon, and rectosigmoid junction

^g^Five cases of mixed type were included

^h^TNM stage was determined based on the 7th edition of AJCC/UICC TNM classification. Low- and high-stage indicate TNM stage I/II and III/IV, respectively

All tumor samples and patients were defined and diagnosed, respectively, at the Department of Pathology and Surgery, Wonkwang University Hospital. Each fresh biopsy sample was aliquoted into two or four tubes. The first sub-sample was stored frozen in liquid nitrogen until immunoblot analysis; the second was immediately fixed with formalin, paraffin embedded, then stored for hematoxylin and eosin staining and immunohistochemical staining; the third was immediately homogenized for subcellular fractionation; and for the fourth, RNA was immediately extracted and the RNA was reverse transcripted. The synthesized cDNA sample was stored frozen at −75 °C until real-time polymerase chain reaction (PCR) analysis. All paraffin-embedded tissue samples were sectioned, stained with hematoxylin and eosin and evaluated twice by two pathologists. All histological findings were consistent with the diagnosis, and all were concordant between the two pathologists. All sections of cancer tissue included at least 30 % cancer cells, and none showed light microscopically-detectable degeneration or necrosis. Aneuploidy status was determined using routine clinical laboratory diagnostic methods consisting of propidium iodide staining followed by flow cytometric analysis. The SW1116 cell line (a CEA-producing colon cancer cell line) was obtained from the American Type Culture Collection and used between passage 3 and 8 after they were obtained.

### Immunoblot and semiquantitative analysis of SF proteins and CEA

Tissue samples (40 mg) were homogenized in RIPA buffer containing protease inhibitors using a Bullet Blender homogenizer (Next Advance; Averill Park, NY, USA), and whole-cell lysate was obtained by sequential centrifugations. Proteins (~30 μg) in the whole-cell lysate were separated on 10 % sodium dodecyl sulfate–polyacrylamide gels with SW1116 cell protein (~30 μg). The proteins were transferred onto polyvinylidene difluoride membranes. The membranes were blocked with 5 % skim milk in Tris-buffered saline containing 0.1 % Tween 20 (TBS-T), rinsed, and incubated with the appropriate antibodies in TBS-T containing 3 % skim milk. Excess primary antibody was then removed by washing the membrane four times in TBS-T. The membranes were then incubated with horseradish peroxidase-conjugated secondary antibody (anti-rabbit or anti-mouse). After three washes in TBS-T, bands were visualized using Clarity Western ECL Substrate (Biorad; Hercules, CA, USA) on the FluorChem E System (Protein Simple; Santa Clara, CA, USA). The following primary antibodies were used in the immunoblot analysis of whole- or fractionated cell lysates: anti-HNRNPA1 (dilution 1:2000; cat. sc-32301; Santa Cruz Biotechnology; Santa Cruz, CA, USA); anti-SRSF1 (dilution 1:1000; cat. 324600; Invitrogen; Carlsbad, CA, USA); anti-SRSF3 (dilution 1:1000; cat. RN080PW; MBL; Nagoya, Japan); anti-SRSF5, anti-SRSF6, anti-SRSF7 (dilution 1:1000; cat. HPA 043484, HPA029005, HPA043850; Sigma-Aldrich; St. Louis, MO, USA); anti-CEA (dilution 1:3000; cat. MS-613-P; Thermo Fisher Scientific; Fremont, CA, USA); anti-ACTB (dilution 1:5000; cat. MA5-15739; Invitrogen); anti-poly (ADP-ribose) polymerase (PARP; dilution 1:1000; cat. sc-7150; Santa Cruz Biotechnology); anti-histone H3 (dilution 1:1000; cat. 9715 s; Cell Signaling; Beverly, MA, USA); and anti-prohibitin (dilution 1:1000; cat. AB28172; Abcam; Cambridge, UK).

Then, the optical density of the region of the target molecular weight (±20 %), as described by the respective antibody manufacturers, was analyzed using ImageJ (http://imagej.nih.gov/ij/). Relative protein levels of the samples were determined after normalization to the β-actin band and calibrated using bands from SW1116 cells (a value of 10 was assigned to the SW1116 cell) on each membrane. As presented in previous reports [13–15] and/or manufacturers’ instructions, SRSF1, SRSF6, SRSF7, HNRNPA1, and CEA showed multiple bands in each target molecular weight area. For the five proteins, multiple bands in each target molecular weight area were calculated and the sum of the distinct band/bands was then analyzed for the proteins. Values of 0.2 (HNRNPA1 and CEA), 0.7 (SRSF1), 0.8 (SRSF3, SRSF5, and SRSF7) or 1.0 (SRSF6) were assigned to the undetected bands of target regions based on band density of SW1116 cell lysate.

### Subcellular fractionation and immunoblot

Ten paired [normal mucosa (NM) and cancer] samples (*n* = 20) were fractionated using a modification of the method described previously [16–18]. Tissue samples (70 mg) were homogenized in 800 μL of homogenization buffer [0.25 M sucrose, 10 mM EDTA, 10 mM EGTA, 2 mM MgCl_2_, 20 mM Tris–HCl (pH 7.4) and protease inhibitors]. The homogenized lysate was centrifuged at 1500 × *g* for 5 min at 4 °C, and the resulting pellet, containing nuclei, and the supernatant (post-nuclear supernatant) were separated. Subsequently, the pellet, containing nuclei, was resuspended in nuclear extraction buffer [2.5 % glycerol, 1 mM EDTA, 1 mM EGTA, 1.5 mM MgCl_2_, 0.42 M NaCl, and 20 mM HEPES (pH 7.6)] for 1 h at 4 °C, centrifuged again at 20000 × *g* for 30 min at 4 °C. The supernatant was then collected and referred to as the nuclear extraction fraction. In parallel, the post-nuclear supernatant was centrifuged at 20000 × *g* for 30 min at 4 °C and the resulting supernatant was saved as the cytosol/microsome fraction. The pellet was resuspended in RIPA buffer for 10 min at 4 °C followed by centrifugation at 1500 × *g* for 5 min at 4 °C, and the resulting supernatant was collected and referred to as the membrane/organelle fraction, including intracellular membranes and some plasma membrane. Immunoblot analysis was performed on these three fractions (nuclear extract, membrane/organelle, and cytosol/microsome) as described above.

The resultant three fractions differed in terms of the total protein amounts (membrane/organelle < nuclear extract < cytosol/microsome) for all tissue samples. In addition, the subcellular fraction indicator proteins (PARP, histone H3, and prohibitin) showed inter-individual and intra-individual (cancer vs. NM) variations. Accordingly, to compare the relative SF expression levels for the respective fractions, an equal amount of total protein per lane was loaded.

### Enzymatic immunohistochemical staining

Formalin-fixed, paraffin-embedded tissue was sectioned and placed on slides. Sections were stained using the Discovery XT automated immunohistochemistry (IHC) stainer (Ventana Medical Systems; Tucson, AZ, USA) and Ventana Chromo Map Kit (Ventana Medical Systems) according to the manufacturer’s instructions. The sections were deparaffinized using EZ prep solution; the antigen was retrieved in cell a conditioning solution (CC1; Ventana Medical Systems) under standard conditions (100 °C, 60 min), and endogenous peroxidase was inhibited by treatment with 3 % H_2_O_2_ for 4 min. Then, the sections on slides were incubated with the primary antibody that had been used in the immunoblot analysis (dilution 1:300 for anti-HNRNPA1 and anti-CEA and dilution 1:100 for other antibodies) for 60 min at 37 °C, and then with a secondary antibody (UltraMap anti-RB HRP or UltraMap anti-MS HRP; Ventana Medical Systems) for 28 min at 37 °C. The sections were incubated in diamidobenzidine and H_2_O_2_ for 8 min at 37 °C followed by counterstaining with hematoxylin and treatment with bluing solution. On completion of staining, sections were dehydrated in alcohol, cleared in xylene, and mounted in synthetic resin.

### mRNA quantification using quantitative real-time PCR

Total RNA from 40 mg of tissue sample was isolated using 1 mL of TRIzol (Life technologies; Carlsbad, CA, USA) in accordance with the manufacturer’s instructions. RNA (500 ng) was reverse transcribed using ReverTra Ace qPCR RT Kits (Toyobo; Osaka, Japan). Quantitative real-time PCR was performed in a StepOnePlus Real-Time PCR System (Applied Biosystems; Foster City, CA, USA) using a SYBR Green Realtime PCR Master Mix (Toyobo) in accordance with the manufacturer’s instructions. Each assay was performed in triplicate, and results were normalized to *18S rRNA* levels. Relative mRNA level was calculated using StepOne software v.2.2.2 and calibrated to that of SW1116 cell sample (A value of 10 was assigned to the mRNA level of SW1116 cell) in each batch test.

Primer sequences were as follows: 5'-TGGATTTGGTAATGATGGAA-3' and 5'-TCTCTGGCTCTCCTCTCCTG-3' (*HNRNPA1*); 5'-TGCCTACATCCGGGTTAAAG-3' and 5'-CTGCTGTTGCTTCTGCTACG-3' (*SRSF1*); 5'-TCTTGGAAACAATGGCAACA-3' and 5'-CTCGGGGATCTTCAAATTCA-3' (*SRSF3*); 5'-GAGGCTTTGGTTTTGTGGAA-3' and 5'-CGAGCCCTAGCATGTTCAAT-3' (*SRSF5*); 5'-AAATACGGACCACCTGTTCG-3' and 5'-CTTCACCTGCTTGTCGCATA-3' (*SRSF6*); 5'-CGCTGGCAAAGGAGAGTTAG-3' and 5'-CGAATTCCACAAAGGCAAAT-3' (*SRSF7*); 5'-GTAACCCGTTGAACCCCATT-3' and 5'-CCATCCAATCGGTAGTAGCG-3' (*18S rRNA*).

### Statistical analysis

All group values were non-normally distributed in the Kolmogorov-Smirnov test (*p* < 0.05). Thus, the values were compared using the Mann–Whitney *U*-test (between two groups) or Kruskal-Wallis test (among more than two groups). If values were significantly different in the Kruskal-Wallis test, Conover’s post-hoc tests were performed. Ratios were compared using the chi-square test for comparison of incidences between GC and CRC. Spearman correlations were used to assess relationships among the levels of SF proteins or CEA and levels of SF mRNAs. The DAs of each SF for discriminating between cancer and NM were obtained by constructing receiver operating characteristic curves. The cut-offs for defining cancer were determined by the respective highest Youden indexes. The statistical differences between the DAs were determined using the DeLong method (for same-sample-derived comparison) or a comparison of the areas under independent receiver operating characteristic curves (for two independent sample-derived comparisons).

Data were analyzed with MedCalc version 12.7 (MedCalc Software, Mariakerke, Belgium) and StatsDirect version 2.7.8 (StatsDirect Ltd, Cheshire, UK). A two-tailed *p* value of less than 0.05 was considered statistically significant.

## Results

### Relative protein levels of the SFs and CEA in GC and CRC

**Fig. 1:**
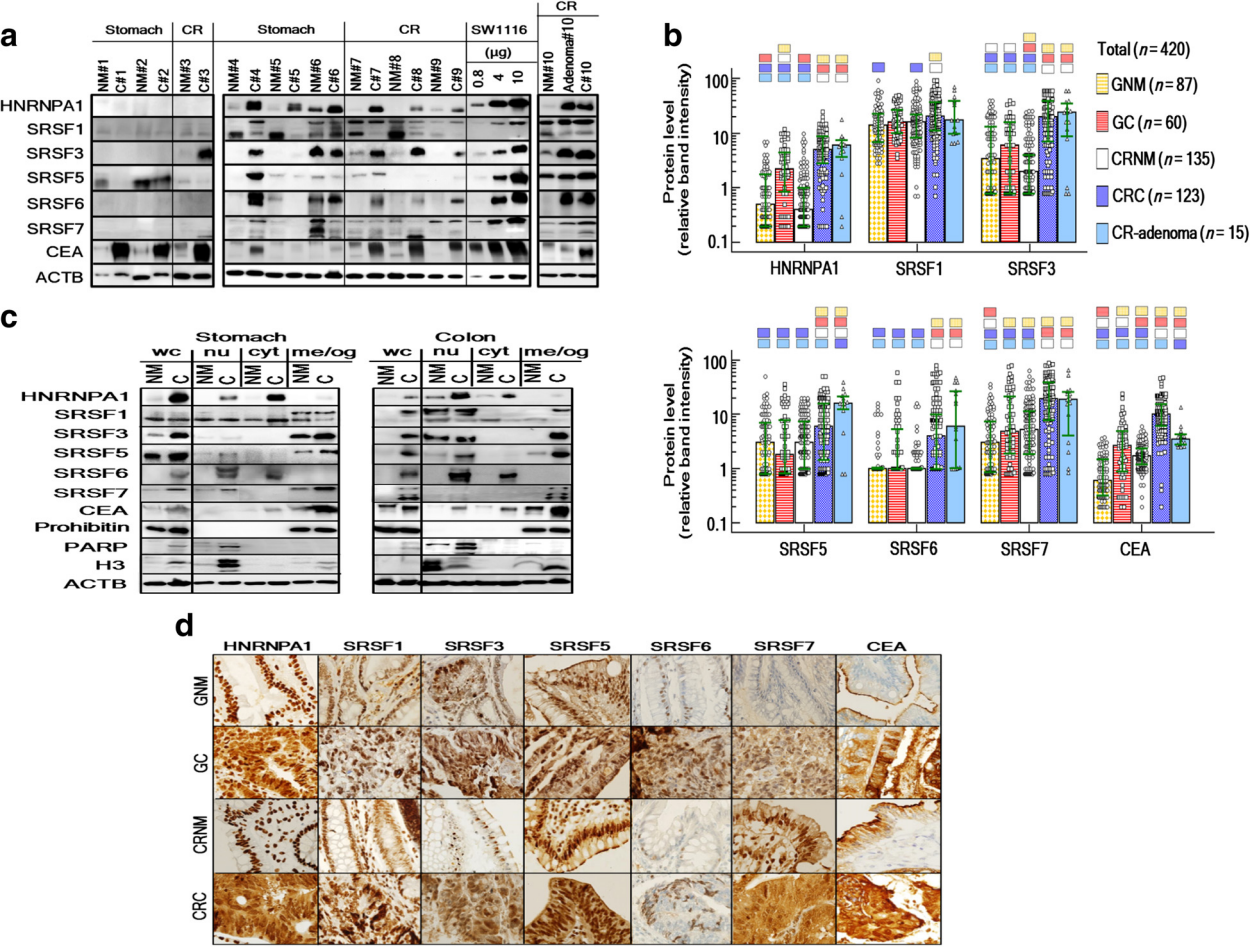
Proteomic expression of six SFs and CEA. **a** Representative semiquantitative immunoblot analysis of gastric and colorectal (CR) cancer (C) and their adjacent normal mucosa (NM) tissue samples. β-actin (ACTB) was used as a loading control. SW1116 cell lysate was loaded as a semiquantitative calibrator. For each SF and CEA, distinct bands in the following target molecular weights were analyzed: 29–39 kDa (HNRNPA1), 27–33 kDa (SRSF1), 20 kDa (SRSF3), 36–40 kDa (SRSF6), 33–15 kDa (SRSF7), and 180–220 kDa (CEA). **b** Relative quantitation of SF proteins and CEA in immunoblot analysis for gastric normal mucosa (GNM), gastric cancer (GC), colorectal NM (CRNM), CR cancer (CRC) and CR-adenoma. Each bar indicates the median and the green horizontal lines indicate the interquartile ranges. The color (compared group) matched small blocks above each median bar indicate the *p* value < 0.05 (by Kruskal-Wallis test followed by Conover’s post-hoc tests). **c** Representative subcellular distribution analysis using biochemical fractionation and immunoblot method. Whole cell (wc); nuclear extract (nu); cytosol/microsome (cyt); and membrane/organelle (me/og) fractions were loaded. The nuclear extract fraction was identified with poly (ADP-ribose) polymerase (PARP) and histone H3 (H3) and membrane/organelle fraction was identified with prohibitin. Respective subcellular fractions were normalized using the total protein quantity/ACTB. The actual experiment involved 10 paired samples (*n* = 20; 5 GC/GNM pairs and 5 CRC/CRNM pairs). **d** Representative immunohistochemical staining analysis. The actual experiment involved 10 paired samples (*n* = 20; 5 GC/GNM pairs and 5 CRC/CRNM pairs)

In a comparison of relative immunoblot band intensity (Fig. 1a and b) of stomach samples, the median levels of HNRNPA1 (2.2 vs. 0.5; 4.4-fold difference; *p* < 0.001), SRSF7 (4.8 vs. 3.0; 1.6-fold difference; *p* = 0.006), and CEA (2.6 vs. 0.6; 4.3-fold difference; *p* < 0.001) were significantly higher in GC samples than in gastric NM (GNM) samples (Fig. 1b). Other SF protein median levels were not significantly (*p* > 0.05) different between GC and GNM samples.

In immunoblot analyses of CR samples (Fig. 1a and b), the median levels of all six SFs and CEA were significantly higher in CRC than in CRNM. Also, the median levels of all SFs except SRSF1 were significantly higher in CR-adenoma than in CRNM. HNRNPA1 and SRSF3 median levels in CRC were most markedly higher than in CRNM samples among the SFs. Median levels of the six SFs were not lower or even higher in CR-adenoma samples compared with CRC samples, but the median level of CEA was lower in CR-adenoma than in CRC.

When GC and CRC were compared (Fig. 1b), median levels of all SFs except SRSF1 were significantly higher in CRC than in GC samples.

### Association between the clinicopathologic factors and SF protein levels

In an association analysis between the clinicopathologic factors and SF proteins (Table 1), none of the SF protein levels were significantly different based on patient age, gender, or cancer location in both cancers. Most GCs and CRCs were adenocarcinomas; in gastric adenocarcinoma, the median level of SRSF7 (7.0 vs. 2.9; *p* = 0.049) was significantly higher in intestinal-type than in diffuse-type. In CR-adenocarcinoma, the median level of HNRNPA1 was significantly higher in well-differentiated type (7.0 vs. 5.0; *p* = 0.020), no lymph node metastasis (7.0 vs. 4.3; *p* = 0.003), or low (≤II)-stage (7.0 vs. 4.3; *p* = 0.003) groups than in the other groups. Median levels of other SF proteins, except HNRNPA1 and SRSF7, were not significantly different based on tumor status, lymph node metastasis, TNM stage, or aneuploidy status in both gastric and CR adenocarcinoma.

### Comparison of upregulation incidences of the SF and CEA proteins in GC and CRC

In paired sample (cancer/NM from each same patient) comparison (Table 2) of stomach samples, only HNRNPA1 showed >50 % upregulation (cancer/NM >2) incidence (UI), followed by the other UIs in the order of HNRNPA1 (52 %), SRSF7 (42 %), and other SF proteins (22 %–33 %). In CRC, all SFs except SRSF1 and SRSF5 showed UIs of >50 % in the order of HNRNPA1 (88 %), SRSF3 (74 %), and other SFs (31 %–57 %). When UIs in GC and CRC were compared, the UIs of all SFs were lower (SRSF3, HNRNPA1, and SRSF6) or tended to be lower in GC than in CRC; the differences between GC and CRC were greatest for SRSF3 (40 %; *p* < 0.001), followed by HNRNPA1 (36 %; *p* < 0.001), SRSF6 (24 %; *p* = 0.004), and the other SFs. The UIs of CEA were >50 % in both cancers; it was also lower in GC (53 %) compared with CRC (92 %), and the results were similar to HNRNPA1 values.

**Table 2:** Comparison of upregulation incidences and detection accuracies of SFs and CEA in GC and CRC

	Protein			mRNA		
	Stomach	Colon and rectum	GC vs. CRC, % difference (*p* ^b^)	Stomach	Colon and rectum	GC vs. CRC, % difference (*p* ^b^)
UI^a^ in paired samples; upregulated pair, *n/*total pair, *n* (%)						
HNRNPA1 or *HNRNPA1*	31/60 (52)	106/121 (88)	36 (< 0.001)	20/45 (44)	55/96 (57)	13 (0.214)
SRSF1 or *SRSF1*	13/60 (22)	38/121 (31)	10 (0.232)	17/45 (38)	37/96 (39)	1 (0.921)
SRSF3 or *SRSF3*	20/60 (33)	89/121 (74)	40 (< 0.001)	20/45 (44)	46/96 (48)	3 (0.838)
SRSF5 or *SRSF5*	15/60 (25)	48/121 (40)	15 (0.074)	16/45 (36)	20/96 (21)	−15 (0.097)
SRSF6 or *SRSF6*	17/60 (28)	63/121 (52)	24 (0.004)	16/45 (36)	46/96 (48)	12 (0.232)
SRSF7 or *SRSF7*	25/60 (42)	69/121 (57)	15 (0.115)	18/45 (40)	45/96 (47)	7 (0.559)
CEA	32/60 (53)	111/121 (92)	38 (< 0.001)	–	–	–
Detection accuracy, AUC						
All^c^ (Sensitivity%/Specificity%)	*n* = 147	*n* = 258		*n* = 111	*n* = 202	
low-stage AC^d^	*n* = 127	*n* = 197		*n* = 95	*n* = 153	
high-stage AC^d^	*n* = 103	*n* = 193		*n* = 78	*n* = 152	
HNRNPA1 or *HNRNPA1*	0.74 (72/71)	0.90^e^ (85/86)	17 (< 0.001)	0.58^e,f^ (69/50)	0.69^e,f^ (54/78)	11 (0.117)
	0.71	0.93	23 (< 0.001)	0.54^e^	0.71^e,f^	17 (0.030)
	0.77	0.89^e^	11 (0.141)	0.64^e^	0.64^e,f^	0 (1.000)
SRSF1 or *SRSF1*	0.58^e,f^ (95/23)	0.62^e,f^ (42/82)	4 (0.487)	0.55^e,f^ (49/65)	0.61^e,f^ (88/35)	6 (0.399)
	0.56^e^	0.66^e,f^	10 (0.147)	0.53^e^	0.62^e,f^	9 (0.252)
	0.66^e^	0.58^e,f^	−8 (0.314)	0.63^e^	0.58^e,f^	−6 (0.552)
SRSF3 or *SRSF3*	0.53^e,f^ (53/57)	0.84^e,f^ (81/78)	32 (<0.001)	0.59^e,f^ (71/52)	0.65^e,f^ (75/51)	7 (0.322)
	0.56^e,f^	0.84^e,f^	28 (< 0.001)	0.54^e^	0.68^e,f^	14 (0.069)
	0.55^e,f^	0.84^e^	29 (< 0.001)	0.60^e^	0.62^e,f^	16 (0.866)
SRSF5 or *SRSF5*	0.51^e,f^ (60/51)	0.62^e,f^ (35/89)	11 (0.062)	0.52^e,f^ (91/23)	0.52^e,f^ (78/38)	0 (0.953)
	0.53^e,f^	0.65^e,f^	12 (0.101)	0.54^e^	0.51^e,f^	−3 (0.718)
	0.62^e^	0.58^e,f^	−3 (0.701)	0.53^e,f^	0.54^e,f^	1 (0.914)
SRSF6 or *SRSF6*	0.61^e,f^ (38/89)	0.76^e,f^ (51/96)	14 (0.006)	0.58^e,f^ (56/62)	0.67^e,f^ (60/68)	9 (0.192)
	0.60^e^	0.78^e,f^	18 (0.004)	0.53^e,f^	0.68^e,f^	15 (0.062)
	0.56^e,f^	0.74^e,f^	18 (0.032)	0.61^e^	0.65^e,f^	4 (0.699)
SRSF7 or *SRSF7*	0.63^e^ (30/84)	0.74^e,f^ (59/83)	11 (0.066)	0.54^e,f^ (38/80)	0.65^e,f^ (85/43)	11 (0.125)
	0.61^e^	0.75^e,f^	15 (0.036)	0.53^e^	0.66^e,f^	12 (0.126)
	0.71^e^	0.73^e,f^	3 (0.755)	0.70^e^	0.64^e,f^	−6 (0.474)
CEA	0.76 (57/89)	0.96^f^ (92/94)	20 (< 0.001)	–	–	–
	0.77^g^	0.97	20 (< 0.001)	–	–	–
	0.90^g^	0.99^f^	9 (0.037)	–	–	–

*Abbreviations*: *AC* adenocarcinoma, *AUC* area under the curve, *CEA* carcinoembryonic antigen, CRC colorectal cancer, GC gastric cancer, *NM* normal mucosa, *UI* upregulation incidence

^a^UIs in cancer were acquired using paired samples from the same patient (cancer/NM >2-fold)

^b^ *p* value was acquired by chi-square test

^c^AUCs were acquired for all types of cancer (including non-AC), regardless of TNM stage

^d^AUCs were acquired for low-stage AC (TNM stage I/II AC) or for high-stage AC (TNM stage III/IV AC)

^e^ *p* < 0.05: compared with the respective AUC of CEA; *p* values for the paired AUC were acquired by the DeLong test

^f^ *p* < 0.05: compared with the respective AUC of HNRNPA1; *p* values for the paired AUC acquired by the DeLong test

^g^ *p* < 0.05: AUC of low-stage AC vs. AUC of high-stage AC; *p* values were acquired by independent receiver operating characteristic curve comparison test

### Comparison of DAs of the SF and CEA proteins in GC and CRC

In the DA analysis (Table 2) for GC, only DA-HNRNPA1 (74 %) was acceptable (>70 %), while the other SFs presented poor (≤70 %) DAs. The DAs of all SFs were not significantly different between low- and high-stage gastric adenocarcinoma. In CRC, all SFs except SRSF1 and SRSF5 presented acceptable DAs in the order of HNRNPA1 (90 %), SRSF3 (84 %), and the other SFs (62–76 %). When the DAs for GC and CRC were compared, the DAs of all SFs were lower (SRSF3, HNRNPA1, and SRSF6) or tended to be lower in GC than in CRC; the differences between GC and CRC were greatest for SRSF3 (32 %; *p* < 0.001), followed by HNRNPA1 (17 %; *p* < 0.001), SRSF6 (14 %; *p* = 0.006), and the other SFs. The DAs of all SFs were not significantly different based on the stage of CR-adenocarcinoma, and the greatest difference between GC and CRC being in SRSF3 was consistently observed regardless of the stage. In the comparison with CEA (Table 2), DA-HNRNPA1 was not different from DA-CEA in both low- and high-stage gastric adenocarcinoma, whereas in CRC, DA-HNRNPA1 was not different from DA-CEA in low-stage CR-adenocarcinoma, but it was significantly lower than DA-CEA in high-stage CR-adenocarcinoma.

### Subcellular distribution of SF proteins in GC and CRC

Previously, several studies showed SF distribution using enzymatic IHC [7].

Generally, this method provides information about both cellular morphology and tissue structural changes, but it is limited when used for a clear discrimination of organelles (the major location of translation regulatory molecules) from cytosol. So far, no study has demonstrated the SF subcellular distribution in primary cancer cells using biochemical fractionation and immunoblotting. Using the biochemical fractionation approach (Fig. 1c), we were able to more precisely distinguish the extranuclear fractions. HNRNPA1 and SRSF6 in both cancers frequently (≥50 %) showed different distribution patterns from those of the other SFs. When HNRNPA1 and SRSF6 were upregulated in whole cancer-cell lysates, they were predominantly distributed in nuclear and/or cytosol/microsome fractions; the upregulated SFs in the cytosol/microsome fraction were detectable in >50 % of upregulated cases in both cancers. Alternatively, when SRSF1, 3, 5, and 7 were upregulated in whole cancer-cell lysates, they were predominantly distributed in nuclear and/or membrane/organelle fractions; the upregulated SFs in the membrane/organelle fraction were detectable in >50 % of upregulated cases in both cancers. There were no remarkable differences in subcellular distributions between GC and CRC. With regard to methodological principle and analytical points, biochemical fractionation followed by immunoblotting differed from IHC. The former was normalized using equal amounts of total protein per lane, while IHC images were analyzed directly. During the former method process, the nuclear and extranuclear fractions were transiently treated in a different buffer, which may affect the affinity of the antibody, and a certain amount of the protein in the border zone of the different fractions was discarded to prevent contamination of a adjacent fraction. Regarding these concerns, we only were able crudely to match the nuclear and extranuclear fraction quantities, and were not able to precisely match the distribution of four SF proteins (SRSF 1, 3, 5, and 7) in the membrane/organelle fraction to the corresponding fraction of the enzymatic IHC analysis (Fig. 1d). Nonetheless, the two methods showed an approximate match with regard to whole-cell SF protein intensities.

### Relative mRNA levels of the SFs in GC and CRC

**Fig. 2:**
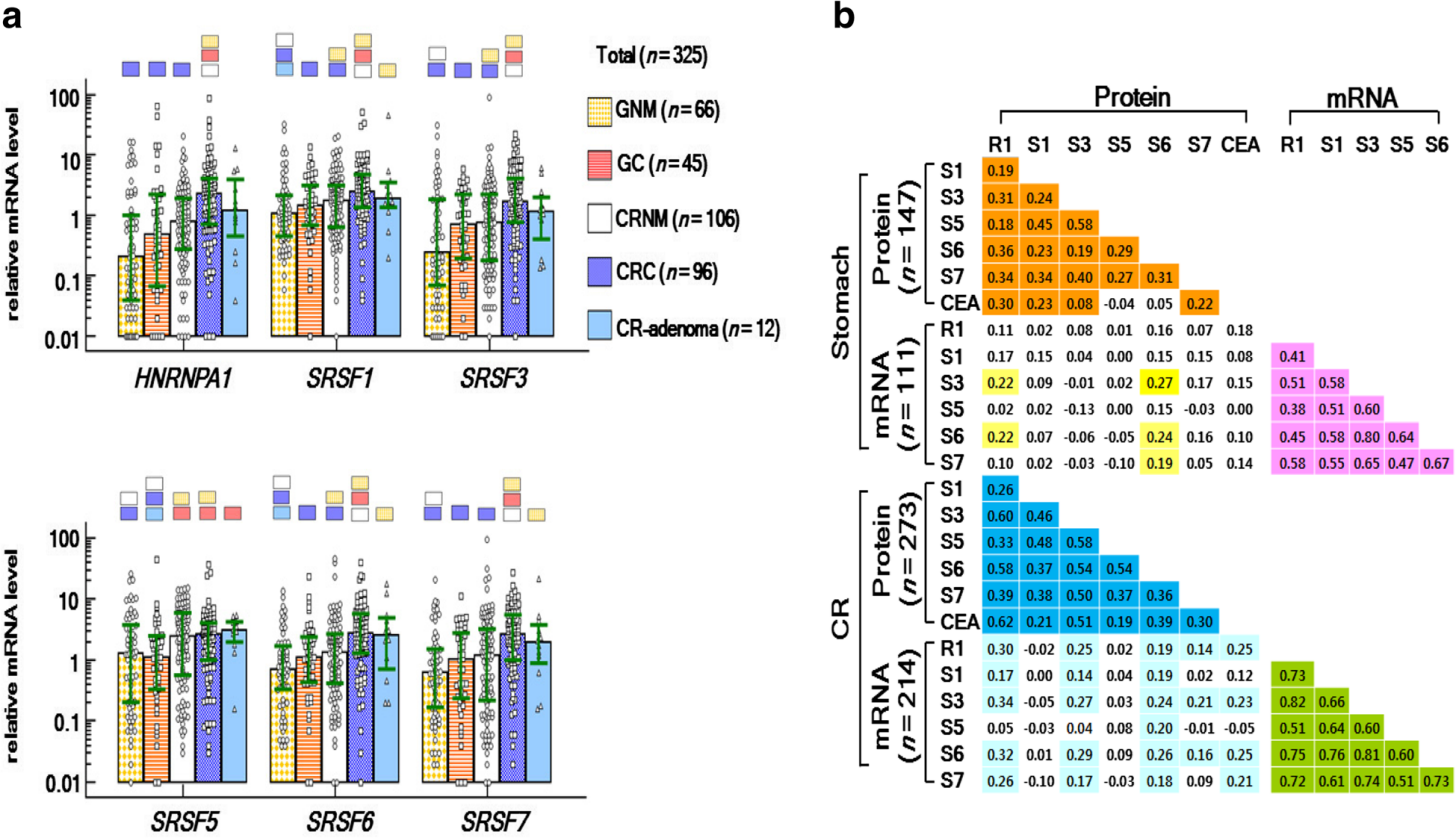
Relative mRNA levels of six SFs (**a**) and correlation analysis among the SF proteins and mRNAs (**b**). **a** Each bar indicates the median, and the green horizontal lines indicate the interquartile ranges for gastric normal mucosa (GNM), gastric cancer (GC), colorectal NM (CRNM), CR cancer (CRC), and CR-adenoma. The color (compared group) matched small blocks above each median bar indicate the *p* value < 0.05 (by Kruskal-Wallis test followed by Conover’s post-hoc tests). **b** R1, S1, S3, S5, S6, and S7 indicate HNRNPA1, SRSF1, SRSF3, SRSF5, SRSF6, and SRSF7, respectively. The numbers in colored cells (*p* < 0.05) or uncolored cells (*p* > 0.05) indicate the correlation coefficient (r)

In the SF mRNA level analysis (Fig. 2a), none of the six SF median levels significantly differed between GC and GNM. In contrast, all SFs except *SRSF5* were significantly higher in CRC than in CRNM.

None of the SF mRNA median levels were significantly different based on histological features, lymph node metastasis, or TNM stage in gastric or CR-adenocarcinoma (Table 1).

In paired sample comparisons of mRNA (Table 2), all SF mRNAs showed <50 % UI in GC, and only *HNRNPA1* showed >50 % UI in CRC.

In DA analysis of mRNA (Table 2), including DA-*HNRNPA1* (69 %) for CRC, which was the highest SF mRNA DA, all of the SF mRNAs showed poor DAs for both cancers. The UIs and DAs of all of the SF mRNAs were not significantly different between GC and CRC (Table 2).

### Correlation between the SF proteins and their respective mRNA levels

In correlation analysis (Fig. 2b), all SF proteins were significantly correlated with each other and all SF mRNAs were correlated with each other in both stomach and CR samples.

None of the SF protein levels, except SRSF6, were correlated with their respective mRNA levels in stomach samples. Whereas in CR samples, levels of three SF proteins (HNRNPA1, SRSF3, and SRSF6), which had relatively high DAs, were correlated with their respective mRNA levels.

## Discussion

Our study demonstrated, for the first time, the following: (1) HNRNPA1 is the most useful, CEA-comparable marker for GC among the six SF proteins and their mRNAs, and HNRNPA1 and SRSF7 have significantly elevated levels in GC tissue compared with GNM; (2) comparative diagnostic priority of HNRNPA1 for CRC is dependent on stage (i.e., DA-HNRNPA1 for CR-adenocarcinoma is comparable to that of CEA in low-stage, but inferior to high-stage, CR-adenocarcinoma) (3) unlike in GC, SRSF3 presents relatively high UI and very good DA in CRC; and (4) extranuclear distribution patterns of HNRNPA1 and SRSF6 (cytosol/microsome) differ from those of the other SFs (membrane/organelle) in both cancers.

Although strong evidence supports the role of several SFs in tumorigenesis [1–4, 14] and suggests their potential as diagnostic markers of cancers [11, 19], to apply the SFs in clinics, identification of their DAs and specific characteristics in the target organ is essential. In this regard, we concurrently compared the six SFs and found that only the levels of SRSF7 and HNRNPA1 are statistically significantly higher in GC than in GNM. Currently, no reports have shown, with statistical significance, elevated levels of SRSF7 in any type of primary cancer or elevated levels of HNRNPA1 in primary GC samples. Our results present robust evidence for the relationship between two SF proteins and GC using a statistically valid sample number. In addition, we found significantly higher levels of six SF proteins in CRC than in CRNM. Previously, a study reported amplified *SRSF6* expression in primary CRC [3], but the data was limited to the gene level. It is difficult to find any report that shows significantly elevated levels of the five SRSF proteins in primary CRC. Here, we provide new evidence of a positive relationship between the five SRSFs and CRC in terms of the protein levels using primary CRC samples.

Even though most GC and CRC originate from gastrointestinal tract mucosa and consist of adenocarcinomas, when the SF protein levels were compared between GC and CRC, CRC generally tended to show higher levels than GC. In addition, UI and DA analyses demonstrated significant differences between GC and CRC for HNRNPA1, SRSF3, and SRSF6, and the difference in SRSF3 was most marked. Although it is difficult to pinpoint the exact reason for this, we postulated that it is related to the previously known different etiologies and pathogenetic factors of GC and CRC [20–22]. These different findings for GC and CRC also hold true for our CEA levels in GC and CRC.

None of the SRSF 5–7 and HNRNPA1 proteins showed significant relationships with unfavorable histopathologic or aneuploidy status [23] in gastric adenocarcinoma or CR-adenocarcinoma. Instead, SRSF7 in gastric adenocarcinoma was higher in intestinal-type, which has a higher survival rate than diffuse-type [24], and HNRNPA1 levels in CRC were higher in well-differentiated, without nodal involvement, or low-stage groups. Furthermore, all SF protein levels in CR-adenoma were either not different from or even higher than those of CRC, which has a poorer prognosis than adenoma. Collectively, the elevated levels of all SFs, especially the two SF proteins, do not seem to correlate with poor prognostic factors in either cancer. For the association between HNRNPA1 and prognostic factors of CRC, previous reports showed contradictory findings. One presented a higher upregulation incidence of *HNRNPA1* in low-stage group than in high-stage group [25], while another [11] presented a lower survival rate in patients who had higher HNRNPA1 levels in CRC tissue. Our HNRNPA1 result was in line with the former finding. As a concurrent comparison study of the diagnostic performance levels of multiple SF proteins, our study was designed in a cross-sectional view for selection of a diagnostic marker rather than a longitudinal view for identification of a prognostic/predictive marker, which generally requires a 3–5-year follow-up period. In this respect, although we do not formally report inconclusive results at this point, we believe that our results are also in line with the former one, since in our preliminary survival analysis for 0.6–2.5 years, patients with higher HNRNPA1 levels tended to have longer relapse-free intervals and overall survival times.

Considering the UIs and DAs among the six SFs, HNRNPA1 was the most effective marker for both cancers. Analytical results in many cancer databases, including The Cancer Genome Atlas (TCGA) data [26, 27], partly supported our findings. Specifically, the SF mRNA median levels in the TCGA data (<1.9-fold) were only marginally upregulated in both cancers as in our mRNA results. In contrast, in this study, HNRNPA1 protein levels presented remarkably elevated levels (>4.4-fold) in both cancers. Together, not only the direct comparison within our data (protein vs. mRNA) but also the indirect comparison with the results of the cancer database (marked fold change of HNRNPA1 in this study vs. marginal fold change of *HNRNPA1* in the database), to a degree, validated the relative excellence of HNRNPA1 compared with *HNRNPA1*. In addition, because the DA-HNRNPA1 for GC and low-stage CRC were comparable with the DA of CEA, one of the most commonly used tumor markers for gastrointestinal cancers in clinical practice, our results indicate that HNRNPA1 is applicable as a novel GC marker as well as an early CRC marker. However, in high-stage CR-adenocarcinoma, DA-HNRNPA1 was significantly inferior to DA-CEA. For this, we assumed that HNRNPA1 production reaches a plateau during the premalignant period in the course of CRC progression. Alternatively, it may be due to microenvironmental factors or the instability of HNRNPA1 in a large tumor mass. Among the SF proteins, SRSF7 in GC and SRSF3 in CRC presented the second highest UI and DA, respectively. However, these were less reliable detection markers of both cancers than CEA.

Although the aberrant cytoplasmic localization of HNRNPA1 has already been observed in CRC tissue [10], subcellular distribution analyses of SRSF3 and SRSF 5–7 in primary cancer cells are rare in the literature, and no studies further describe the extranuclear fractions (cytosol/microsome vs. membrane/organelle). We employed a pilot approach using biochemical fractionation and immunoblot assay for the SF distribution analysis in GC and CRC tissue. We detected aberrantly upregulated proteins in the extranuclear fractions in both types of cancer cells and noted that the major distribution patterns of proteins in the extranuclear fractions differ based on the SF type [HNRNPA1 and SRSF6 (cytosol/microsome) vs. SRSF1, 3, 5, and 7 (membrane/organelle)] rather than by cancer type (GC vs. CRC). The cytoplasmic expression of SFs in our IHC analysis, especially prominent in cancer samples, could be explained by two plausible mechanisms: (1) tumor-specific translation control by SFs and (2) tumorigenesis-prone or tumor-associated conditions. Regarding the first mechanism, SFs interact with translation regulators (tumor-promoting or -suppressing factors), which are mainly found in cellular organelles (membrane/organelle fraction) and cytosol (cytosol/microsome fraction) [5, 6]. The second mechanism is associated with the post-translational modification of SFs; rapidly growing tumor cell-related or tumorigenesis-prone stress/status (e.g., metaphase, hypoxia or ultraviolet light) promotes the phosphorylation-related extranuclear localization and/or induction of SF proteins [5, 7, 8].

Because HNRNPA1 and SRSFs have a well-known antagonistic relationship [9], we expected an inverse correlation between HNRNPA1 and SRSF proteins, but all of the SF proteins showed positive correlations with each other. All of the SF mRNAs also showed positive correlations with each other in GC or in CRC. This may be due to collapse of the normal homeostatic relationship among the SFs in cancer cells and/or serial escalation within the SF proteins or within the SF mRNAs. Meanwhile, five SF proteins in GC and three SF proteins in CRC showed expression levels disproportional to those of their respective mRNAs. This could be explained by the specific kinetics of each SF or various post-transcriptional modifications [28–30] that affect the SF protein production rate or stability.

The major purpose of this study was to select an effective marker for GC. Through the comparison of proteins and mRNAs, aside from selection of a specific SF, our results indicate that the HNRNPA1 protein itself, rather than its mRNA, is the most reliable tool for GC detection among the SFs tested here.

## Conclusions

Apart from indicating that among the six SFs, HNRNPA1, but not *HNRNPA1* mRNA, is the most clinically applicable marker for GC, our results offer new insight into the expression profiles and diagnostic efficacies of the six SFs in GC that is required for their future practical implementations. Despite the frequent upregulation and very good to excellent detection accuracy of SRSF3 and HNRNPA1 in CRC, we still recommend the use of CEA, rather than the SFs, especially for high-stage CRC detection.

## Abbreviations

CEA, carcinoembryonic antigen; CR, colorectal; CRC, colorectal cancer; CRNM, colorectal normal mucosa; DA, detection accuracy; GC, gastric cancer; GNM, gastric normal mucosa; HNRNPA1, heterogeneous nuclear ribonucleoprotein A1; IHC, immunohistochemistry; NM, normal mucosa; PCR, polymerase chain reaction; SF, splicing factor; SRSF, serine/arginine-rich splicing factor; TBS-T, Tris-buffered saline containing 0.1 % Tween 20, TCGA, The Cancer Genome Atlas; UI, upregulation incidence.
